# Sirt6 Deacetylase: A Potential Key Regulator in the Prevention of Obesity, Diabetes and Neurodegenerative Disease

**DOI:** 10.3389/fphar.2020.598326

**Published:** 2020-12-07

**Authors:** Swapnil Raj, Liston Augustine Dsouza, Shailendra Pratap Singh, Abhinav Kanwal

**Affiliations:** ^1^ Department of Pharmacology, Manipal College of Pharmaceutical Sciences, Manipal Academy of Higher Education, Manipal, India; ^2^ Department of Biomedical Engineering, School of Engineering and Technology, Central University of Rajasthan, Kishangarh, India; ^3^ Department of Pharmacology, All India Institute of Medical Sciences (AIIMS), Bathinda, India

**Keywords:** drug discovery, epigenetics, diseases, modulators, Sirt6, sirtuins

## Abstract

Sirtuins, NAD + dependent proteins belonging to class III histone deacetylases, are involved in regulating numerous cellular processes including cellular stress, insulin resistance, inflammation, mitochondrial biogenesis, chromatin silencing, cell cycle regulation, transcription, and apoptosis. Of the seven mammalian sirtuins present in humans, Sirt6 is an essential nuclear sirtuin. Until recently, Sirt6 was thought to regulate chromatin silencing, but new research indicates its role in aging, diabetes, cardiovascular disease, lipid metabolism, neurodegenerative diseases, and cancer. Various murine models demonstrate that Sirt6 activation is beneficial in alleviating many disease conditions and increasing lifespan, showing that Sirt6 is a critical therapeutic target in the treatment of various disease conditions in humans. Sirt6 also regulates the pathogenesis of multiple diseases by acting on histone proteins and non-histone proteins. Endogenous and non-endogenous modulators regulate both activation and inhibition of Sirt6. Few Sirt6 specific non-endogenous modulators have been identified. Hence the identification of Sirt6 specific modulators may have potential therapeutic roles in the diseases described above. In this review, we describe the development of Sirt6, the role it plays in the human condition, the functional role and therapeutic importance in disease processes, and specific modulators and molecular mechanism of Sirt6 in the regulation of metabolic homeostasis, cardiovascular disease, aging, and neurodegenerative disease.

## Introduction

**FIGURE 1:**
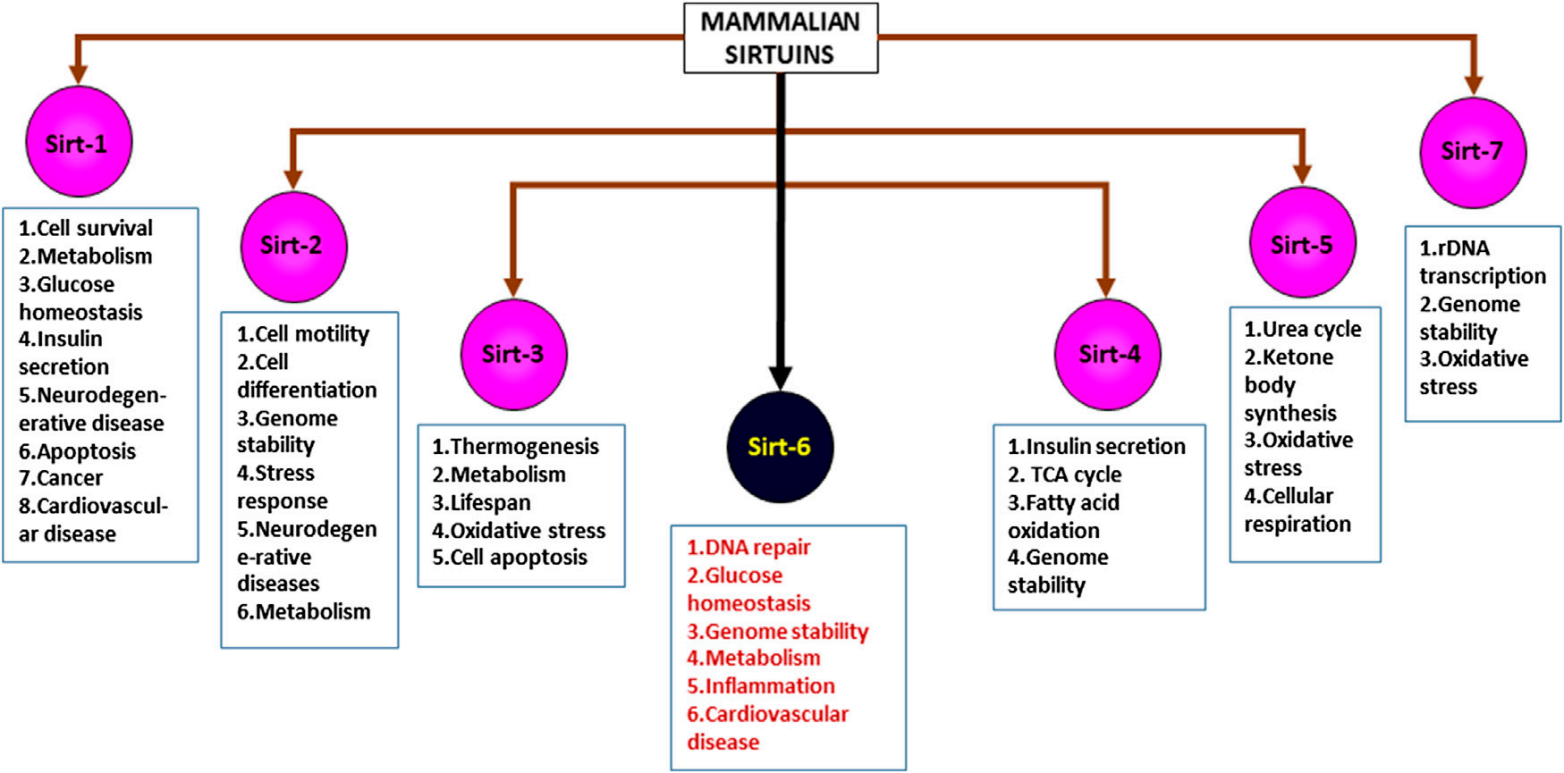
Mammalian sirtuins and their functions.

**FIGURE 2:**
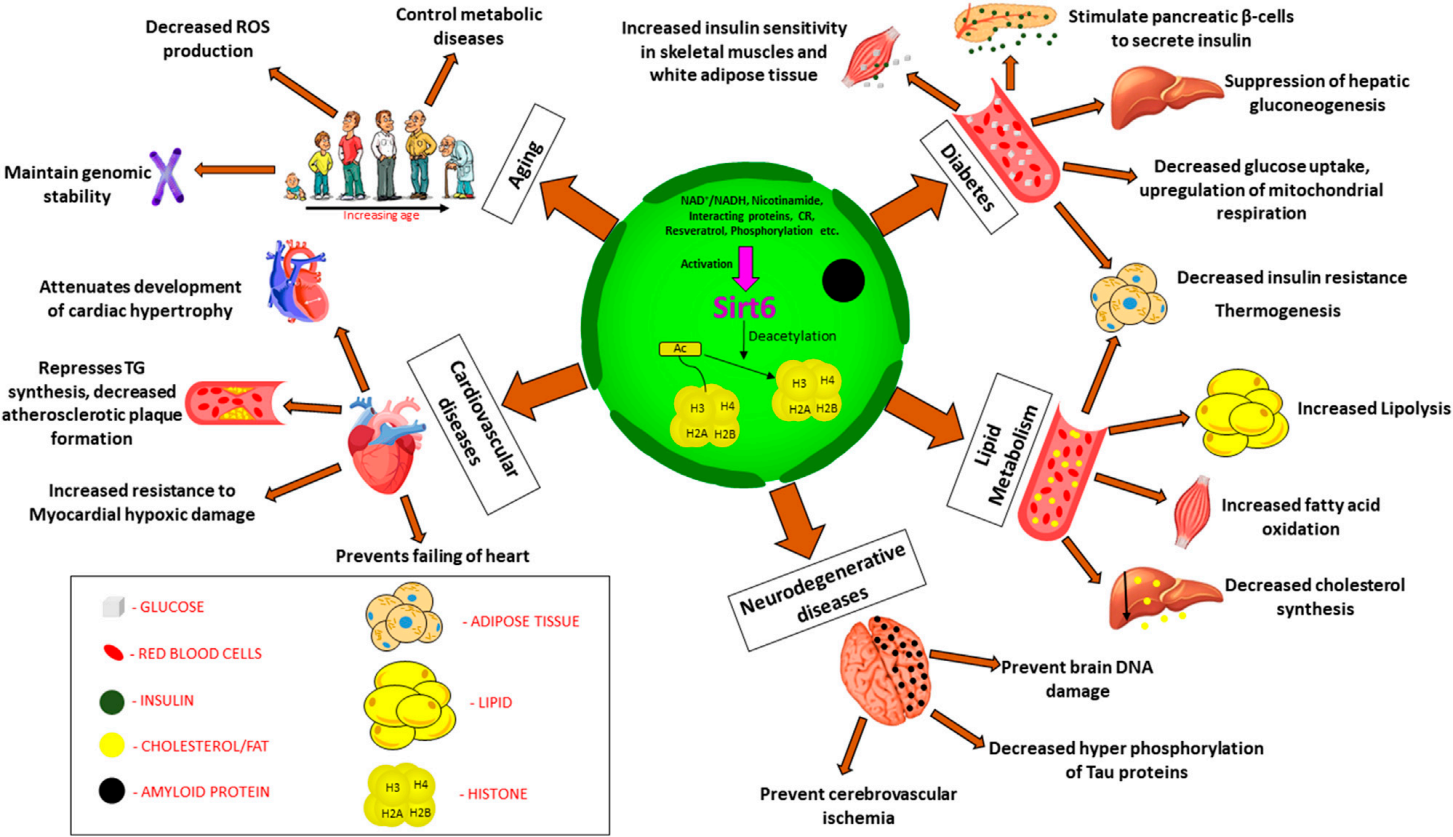
Clinical significance of Sirt6 in various diseases. Sirt6 is a nuclear sirtuin activated by CR, NAD+, resveratrol etc., promoting deacetylation of histone which plays a major role in (a) Diabetes: increased insulin sensitivity, insulin secretion, suppress gluconeogenesis, decrease glucose uptake. (b) Lipid metabolism: increased lipolysis, increased fatty acid oxidation, decreased cholesterol synthesis. (c) Neurodegenerative diseases: decreased phosphorylation of Tau proteins, prevent cerebrovascular ischemia and brain DNA damage (d) Cardiovascular diseases: decrease TG synthesis, attenuates cardiac hypertrophy, prevents failing of heart, resistance to hypoxic damage (e) Aging: decreased ROS production, maintain genomic stability, control metabolic diseases. Abbreviation: Ac, Acetylated; CR, calorie restriction; TG, triglycerides; ROS, reactive oxygen species.

Sirtuins are energy linked NAD + dependent proteins that are activated during calorie restriction (Corbi et al., 2012; Rack et al., 2015; Bheda et al., 2016; Yu et al., 2018), mainly altering the acetylation/deacetylation status of histones, thus regulating chromatin silencing. Proteomic studies suggest that human cells possess histones with various lysine acyl modifications and these sirtuins have specific deacetylating activities that remove acyl moieties from lysine, thus modulating gene expression, without altering the gene sequence itself (Kupis et al., 2016). This unique property of sirtuins and their ability to bind to various substrates and other moieties bearing acyl modifications allow us to examine different mechanisms involved in multiple diseases, thus showing the importance of sirtuins (Carafa et al., 2012). Sirtuins are epigenetic regulators, belonging to class III Histone Deacetylases (HDACs) (Rack et al., 2015) which are known to affect multiple pathways involved in various disease conditions including cancer, diabetes, cardiac failure, hypertrophy, cachexia, pulmonary fibrosis, aging etc. (Dryden et al., 2003; Vassilopoulos et al., 2011; Lee and Goldberg, 2013; Lee and Gu, 2013; Bheda et al., 2016; Kupis et al., 2016; Graham et al., 2018; Kanwal and Dsouza, 2019) These energy linked proteins were first discovered in the yeast cells, as silencing information regulator-2 (Sir-2) (Haigis and Sinclair, 2010; Grabowska et al., 2017; Graham et al., 2018; Rajabi et al., 2018). Orthologues of Sir-2 in mammals are known as sirtuins (Polito et al., 2010). In humans, sirtuins are divided into seven types (Figure 1, Mammalian sirtuins and their functions) (Sirt1- Sirt7), localized in different cellular components, that has crucial role in various cellular processes (Kobayashi et al., 2005; Haigis and Sinclair, 2010; Vassilopoulos et al., 2011; Hoffmann et al., 2014; Wu et al., 2015; Tang, 2016; Dai et al., 2018; Graham et al., 2018; Wang et al., 2018; Kanwal, 2018). Significant differences exist between sirtuin homologues in their variable N and C terminal extensions with conserved C-terminal extensions. Among the 7 types of sirtuins, Sirt6 is an essential sirtuin in humans that is localized in the nucleus. Like other sirtuins, Sirt6 is also a stress responsive protein deacetylase, but unlike other sirtuins, in addition to its deacetylase activity, it also transfers ADP ribosyl via mono-ADP ribosyltransferase enzyme. In humans, Sirt6 has a plethora of functions, including DNA repair, telomerase function, genomic stability, cellular senescence, and metabolic homeostasis (Figure 2: Clinical significance of Sirt6 in various diseases) (Michishita et al., 2008; Watroba and Szukiewicz, 2016). Until recently, Sirt6 was mainly known for chromatin signaling. Recent data indicate that Sirt6 is involved in various disease conditions mentioned above (Khan R. I. et al., 2018); due to their action on multiple substrates and catalytic sites (Tasselli et al., 2017). Studies indicate that they control activities of p53, FOXO proteins, NF-KB, PGC-1α, PARP1, TNFα, GCN5, HIF1α, which are involved in the pathogenesis of various diseases (Kawahara et al., 2009; Dominy et al., 2012; Zwaans and Lombard, 2014). This review focuses on Sirt6, its mechanism(s) in multiple disease states, its significance in human health, and as a therapeutic target in drug discovery.

## Sirt6: Importance and Recent Development

Sirt6 is a NAD^+^ dependent nuclear histone deacetylase, having deacetylase, deacylase and mono-ADP ribosyltransferase activity. In the past 5 years, the development of Sirt6 suggests a crucial role in a broad spectrum of metabolic processes. Sirt6 has a significant role in maintaining genetic stability and DNA repair, by activating several DNA-repair genes (Mao et al., 2011; Khan R. I. et al., 2018). It was identified as a suppressor of genomic instability due to its association with chromatin through its ability to modulate base excision-pair repair and double strand break repair (Kugel and Mostoslavsky, 2014). In addition to maintaining genetic stability, it also plays a crucial role in intermediary metabolism including glucose metabolism, lipid metabolism, circadian metabolism, etc. (Watroba and Szukiewicz, 2016; Tasselli et al., 2017). Studies in the recent past have also shown implications of Sirt6 in various diseases including dyslipidemia, diabetes, heart disease, cancer, neurodegenerative diseases, brain aging etc. (Roichman et al., 2016; Xiwen et al., 2016; D’Onofrio et al., 2017). Sirt6 is a longevity protein; various studies have demonstrated its involvement in increasing life expectancy (Schumacher, 2011; Kanfi et al., 2012; Giblin and Lombard, 2016; Schumacher, 2017). Overexpression of Sirt6 in transgenic mice resulted in significantly longer half-life compared to wild-types, and that IGF-1 plays a crucial role in regulating lifespan (Kanfi et al., 2012). Sirt6 blocks the action of IGF-1, which is responsible for increasing lifespan (Sundaresan et al., 2012). There are two main theories that postulate how Sirt6 regulates longevity, they are: maintenance of genetic stability and regulation of metabolism, and various studies show that either of these theories contribute to Sirt6’s function in longevity (Mostoslavsky et al., 2006; Michishita et al., 2008; Kanfi et al., 2012). Sirt6 knockout mice died within a month of birth due to the development of significant metabolic abnormalities due to deficiency of IGF-1 (Watroba and Szukiewicz, 2016). Lack of IGF-1 was correlated with a decrease in adipose tissue, lordokyphosis, and severe hypoglycemia (Lombard et al., 2008; Peshti et al., 2017; Ferrer et al., 2018). Another study reveals the role of Sirt6 in longevity by the maintenance of genetic stability (Tian et al., 2019). The mechanism involved is double-strand break repair of DNA; an activity controlled by Sirt6 resulted in an increase in lifespan in animal models (Mostoslavsky et al., 2006; Lombard et al., 2008; Tian et al., 2019). Macaque monkeys that lacked the gene for Sirt6 died a few hours after the birth and exhibited prenatal developmental retardation (Zhang W. et al., 2018). In this study, it was observed that the brain was underdeveloped, suggesting low levels of Sirt6 can lead to neurodegenerative diseases and brain aging (Zhang W. et al., 2018; Naiman and Cohen, 2018; Niu, 2019). Sirt6 is necessary in human development as shown by a study that the deletion of Sirt6 in humans can cause perinatal lethality (Ferrer et al., 2018). Several other studies have highlighted telomeres’ role in aging and its association with Sirt6 (Aubert and Lansdorp, 2008; Tennen et al., 2011). With aging, the length of the telomere declines, but Sirt6 by its deacetylation activity, maintain the length of telomeres, thus preventing telomere sequence loss (Aubert and Lansdorp, 2008; Cacchione et al., 2019; Cao et al., 2019). Cells lacking Sirt6 had malformed telomere structure and also replication associated sequence loss of telomeres (Aubert and Lansdorp, 2008). Apart from telomeres, mammalian aging and aging related disorders are also associated with abnormal IGF-Akt signaling, which in turn is controlled by Sirt6 (Pillai et al., 2014). Heart failure is one of the age-related disorder in mammals, where the IGF-Akt on sustained activation promotes hypertrophy and heart failure (Wang et al., 2015; D’Onofrio et al., 2017; Lee and Kim, 2018), whereas Sirt6 impedes IGF-Akt signaling via c-Jun by deacetylation of H3K9 (Sundaresan et al., 2012). Few studies found that failing hearts of humans and mice showed decreased levels of Sirt6, suggesting that low levels of Sirt6 increase the activity of IGF-Akt, leading to initiation and progression of cardiac hypertrophy and heart failure (Sundaresan et al., 2012; Zhang D. et al., 2018). Osteoporosis is yet another age-related disorder in mammals, and recent studies have found that Sirt6 prevents osteoporosis, but the mechanism remains unclear. Sirt6 knockout mice suffering from osteopenia, exhibited more significant bone loss than the non-mutant mice (Zhang D. et al., 2018), indicating that Sirt6 was involved in decreased osteoclast activation (Zhang D. et al., 2018; Wang and Mbalaviele, 2019).

## Substrates for Sirt6

Sirt6 functions are diversified (Pan et al., 2011; Kuang et al., 2018). They include different molecular pathways associated with glycolysis, DNA repair, gluconeogenesis, cardiac hypertrophic responses, neurodegeneration, and tumorigenesis due to its activity on varied number of substrates, which include PARP1, TNFα, GCN5, HIF1α, etc. (Liu et al., 2012; Sundaresan et al., 2012; Li et al., 2017; Kuang et al., 2018; Khan R. I. et al., 2018; Yang H. et al., 2019). HIFs are transcription factors that are expressed as regulators of genes during cellular deprivation of oxygen. Overexpression of HIFs is implicated with tumor growth and metastasis, and is involved in initiating angiogenesis (Jun et al., 2017; Pezzuto and Carico, 2018). Sirt6 can inhibit these activities of HIF. Thus activation of Sirt6 controls tumor growth and metastasis by regulating the overexpression of HIF1α (Zhong et al., 2010; Zwaans and Lombard, 2014; Yang Z. et al., 2019).

TNF-α, a Sirt6 substrate, is a proinflammatory cytokine that is involved in various inflammatory pathological processes (Ravussin and Smith, 2016; Josephs et al., 2018), and increased expression of Sirt6 inhibits TNF-α (He et al., 2017). Although Sirt6 is essential in the deacetylation process, the de-fatty acylation, specifically the hydrolysis of lysine units at the 19 and 20 positions of H3 histone, regulates TNF-α secretion (Jiang et al., 2016). Catalysis of fatty acyl lysine hydrolysis by Sirt6 is more efficient when compared to deacetylation (Jiang et al., 2013). Sirt6 has a significant role in chronic inflammation due to its association with TNF-α. Cells treated with TNF-α had decreased levels of Sirt6 in a dose dependent manner, identifying a symbiotic relationship between TNF-α and Sirt6 (Yeo et al., 2017). In addition to the above, Sirt6 attenuates inflammatory response through inhibition of NF-κB signaling (Li Z. et al., 2018; Santos-Barriopedro et al., 2018; Santos-Barriopedro and Vaquero, 2018).

Sirt6 binds to and activates GCN5, thereby inhibiting the acetylation of PGC-1α (Peroxisome Proliferator activated Receptor coactivator one alpha) and decreasing gluconeogenic gene expression (Giblin and Lombard, 2016; D’Onofrio et al., 2017; Kuang et al., 2018; De Céu Teixeira et al., 2019). This correlation between Sirt6 levels and glucose metabolism is a key in understanding involvement of Sirt6 in the pathogenesis in diabetes mellitus and a potential therapeutic target in the management of the disease (Dominy et al., 2012; D’Onofrio et al., 2017; Kuang et al., 2018).

Sirt6 deacetylase, along with FoxO3a, can reduce LDL-cholesterol levels by regulating the expression of PCSK9 (Proprotein Convertase Subtilisin/Kexin Type 9) (Chaudhary et al., 2017). PCSK9 is a gene that regulates LDL receptors, which in turn is involved in decreased LDL clearance from the circulation in the hepatocytes (Spolitu et al., 2019). FoxO3 recruits NAD^+^ -dependent Sirt6 deacetylase, to the promoter region of the PCSK9 gene for the deacetylating histone H3 at lysine 9 and 56, which resulted in suppression of the expression of PCSK9 gene, thereby promoting LDL receptors and subsequent hepatic clearance of pathogenic LDL (Tao et al., 2013a; Gao et al., 2018). Activation of Sirt6 inhibited PCSK9, thus positively regulating hypercholesterolemia. Therefore Sirt6 could be a potential target in the treatment of dyslipidemia (Tao et al., 2013a; Chaudhary et al., 2017; Glerup et al., 2017; Handelsman and Lepor Norman, 2018; Spolitu et al., 2019).

The activity of Poly (ADP-ribose) polymerase 1 (PARP1) is increased when acted upon by Sirt6 where it undergoes mono (ADP-ribosylation). This enzyme is involved in the modification of nuclear proteins, responsible for DNA repair and proliferation, differentiation and tumor transformation (Xu et al., 2015; Rizzo et al., 2016; Van Meter et al., 2016; Tang, 2017; Fujimoto et al., 2017; Yang et al., 2018). PARP-1 is also an essential regulator of Apoptosis Inducing Factor (AIF) mediated cell death (Song Y. et al., 2016). The expression levels of this protein were low under Sirt6 deficient conditions. Levels of PARP-1 were reduced faster under Sirt6 defective conditions (Zhang Q. et al., 2019).

Recently attention has been focused on the role of Thioredoxin-interacting protein (TXNIP), as overexpression of this protein is negatively associated with the insulin secretion by β-cells of the pancreas (Alhawiti et al., 2017; Nagaraj et al., 2018). Sirt6 influences the expression of TXNIP i.e. overexpression of Sirt6 inhibits TXNIP, thus making it an important therapeutic target (Kuang et al., 2018; Qin et al., 2018). In addition to the above, Sirt6 has been reported to act on various other substrates that could potentially be implicated in multiple other diseases for which a better understanding of the molecular mechanism is required (Tasselli et al., 2017; Khan R. I. et al., 2018; Gertman et al., 2018).

## Sirt6 and Diseases

As we delve into the cellular level, Sirt6 deprivation leads to several changes in sensitivity to reactive oxygen species, glucose metabolism, and genomic stability (Mostoslavsky et al., 2006; Kanfi et al., 2012; Li et al., 2017; Peshti et al., 2017; Xu et al., 2019; Yepuri and Ramasamy, 2019). Mice placed on caloric restriction or Sirt6 activators overexpress Sirt6, improving cancer, and age-related disorders in animal models (Zhang et al., 2016a; Kuang et al., 2018; Iachettini et al., 2018; Rahnasto-Rilla et al., 2018), on the contrary, lower Sirt6 levels in mice showed shorter life expectancy, cancer occurrence, diabetes and other metabolic disorders increased. The animals also exhibited other complications such as curved spines, decreased subcutaneous fat, hypoglycemia, and lowered levels of IGF-1 (Peshti et al., 2017; Khan R. I. et al., 2018; Ghosh et al., 2018; Simon et al., 2019). This suggests that Sirt6 is a therapeutic target in aging, cardiac disorders, neurodegenerative disorders, and metabolic disorders (Rodgers and Puigserver, 2006; Serravallo et al., 2013; Demir et al., 2017; Harlan et al., 2019). Table 1.

**Table 1:** Sirt6: action on various targets in diseases.

DISEASE	TARGET	EFFECT	References
Aging	H3K9ac	Telomere stability, DNA damage response	(Khan R. I. et al., 2018)
	H3K56ac	Telomere stability, DNA damage response	(Khan R. I. et al., 2018)
	H3K18ac	Heterochromatin silencing	(Khan R. I. et al., 2018)
	IGF-1	Reduction in somatotropic axis	(Mao et al., 2018)
Cardiac disorder	PDK4	Improve cardiac glucose metabolism	(Khan D. et al., 2018)
	IL1, NF-ΚB	Inhibit activation of pro-inflammatory cytokines responsible for atherosclerosis; Prevent endothelial damage	(Vitiello et al., 2017)
	ICAM-1, PAI-1	Protect endothelial damage	(Guo et al., 2019)
			(D'Onofrio et al., 2017)
	IGF	Prevent heart failure and cardiac hypertrophy	(Sundaresan et al., 2012)
	Nmnat-II	Activates Nmnat-II, prevent cardiac hypertrophy	(Cai et al., 2012)
Neurodegenerative disease	WRN	Maintains genomic stability and telomeric length	(Khan R. I. et al., 2018)
	GSK3α/β	Decreased tau protein activation	(Tang, 2017)
	Aβ42	Prevents DNA damage	(Jung et al., 2016)
Lipid metabolism	SREBP	Suppresses LDL- cholesterol synthesis	(Kuang et al., 2018)
			(Tao et al., 2013b)
	CK2	Facilitates adipogenesis	(Kuang et al., 2018)
			(Chen et al., 2017)
	PGC1α	Increased Brown adipose tissue thermogenesis and expression of thermogenesis genes	(Kuang et al., 2018)
			(Yao et al., 2017)
	PCSK9	Decreased LDL cholesterol levels	(Kuang et al., 2018)
			(Tao et al., 2013a)
Diabetes	TXNIP	Increased glucose stimulated insulin secretion	(Qin et al., 2018)
	FOXO1	Reduces expression of gluconeogenic genes	(Kuang et al., 2018)
	GLUT1, LDH & PDK1	Promote glycolysis	(Hu et al., 2006)
			(Zhong et al., 2010)
	GCN5 & PGC1α	suppress hepatic gluconeogenesis	(Dominy et al., 2012)

H3K9/56/18ac‐ acetylated histone 3 at lysine position 9, 56, 18 respectively; IGF‐ insulin like growth factor‐1; PDK4‐ pyruvate dehydrogenase kinase 4; IL1‐ Interleukin 1; NF‐KB‐ Nuclear factor kappa B; ICAM1‐ Intercellular adhesion molecule 1; PAI1‐ Plasminogen activator inhibitor 1; Nmnat2‐ Nicotinamide mononucleotide adenylyl transferase; WRN‐ WRN gene that encodes for Werner protein; GSK3α/β‐ Glycogen synthase kinase 3α/β; Aβ42- Plasma amyloid β peptide 42; SREBP- Sterol regulatory element binding protein; CK2‐ Casein kinase 2; PGC1α‐ Peroxisome proliferator activated receptor gamma coactivator 1 alpha; PCSK9‐ proprotein convertase subtilisin/kexin type 9; TXNIP‐ Thioredoxin interacting protein; FOXO1‐ Forkhead box 1; GLUT1‐ Glucose transporter 1; LDH‐ lactic acid dehydrogenase; GCN5‐ General control non depressible 5

## Sirt6 in Aging

Aging is a complex, multifactorial process resulting in the accumulation of diverse harmful changes in the cell, increasing risk of disease and death. Multiple theories can explain the aging process but none can be considered absolute (Tosato et al., 2007; Jin, 2010; Sergiev et al., 2015). These include evolutionary theory, Free radical theory, Mitochondrial theory, Gene regulation theory, Telomere theory, Inflammation hypothesis, Immunity theory, Neuroendocrine theory, Neuroendocrine- immune method theory, and Caloric restriction (Wei et al., 2001; Gavrilov and Gavrilova, 2002; Tosato et al., 2007; López-Lluch and Navas, 2016). In addition to these theories, sirtuins too contribute to aging. Initially, sirtuins were first identified in yeast, which led them to discover its life prolonging activity. Studies in the worm flies confirmed the link between sirtuins and aging (Finkel et al., 2007; Guarente, 2007). Of the seven mammalian sirtuins, Sirt6 is pivotal in regulating lifespan (Kanfi et al., 2012; Hirvonen et al., 2017; Peshti et al., 2017). It promotes chromatin changes essential for DNA repair and maintenance of telomere structure, preventing genomic instability, and cellular senescence (Tasselli et al., 2017). The DNA repair mechanism in longevity is Double-Strand Break repair, where Sirt6 performs this function more efficiently (Tian et al., 2019). Sirt6 is thought to have a protective action on telomeres, as deficiency of Sirt6 causes a loss of telomere sequence associated with replication, leading to genomic instability and early cell death (Watroba and Szukiewicz, 2016; Naiman and Cohen, 2018). Furthermore, Sirt6 maintains redox homeostasis in mesenchymal stem cells, thus suggesting that it regulates longevity (Liao and Kennedy, 2016; Pan et al., 2016). IGF-1 is yet another factor that is related to aging. Lower levels of IGF-1 delayed the process of aging. Transgenic mice models that overexpressed the Sirt6 gene had low levels of IGF-1, slowing the aging process in mice (Kanfi et al., 2012; Ravi et al., 2019). Thus, Sirt6 can be targeted for therapeutic interventions in aging and aging related diseases (Khan R. I. et al., 2018).

## Sirt6 in Cardiac Disorders

**FIGURE 3:**
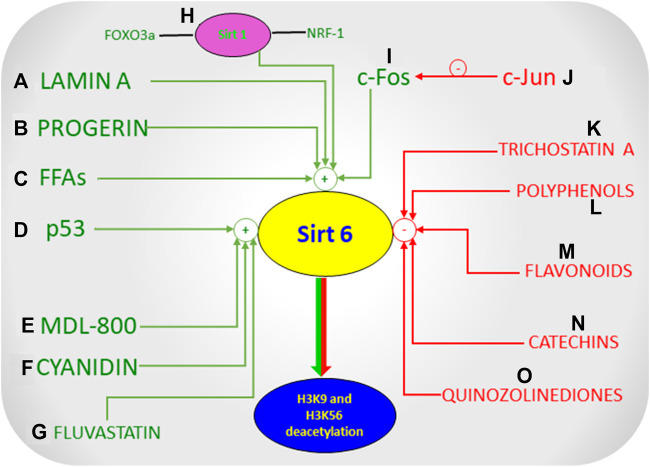
Schematic representation of molecular mechanism of Sirt6 in various diseases. **(A)** Deacetylates H3K9, inhibiting NF-kB and differentiation of cardiac cells into myofibroblasts. Also binds to c-Jun, promoting deacetylation of H3K9, inhibiting the expression of NF-kB and IGF-Akt signalling. Inhibits activation of Ang 2 and STAT3 via Nmnat1 and NAD, together preventing Cardiac Hypertrophy and subsequently Heart Failure. **(B)** Exerts anti-inflammatory action in the endothelial cells via blockade of NF-kB, cytokines (i.e., IL-1, IL-6, IL-8), metalloproteinases (MMP-2, MMP-9), PAI-1, ICAM-1, and COX-2. Delays senescence by inhibiting p21 signalling and maintaining high levels of eNOS. **(C)** Prevents monocyte adhesion and Atherogenesis by inhibiting pro inflammatory mediators (ICAM-1, VCAM-1, MCP-1). Regulates Cholesterol levels by deacetylating H3K9 and inhibiting SREBP gene. **(D)** Enhances activity of GCN5, leading to down-regulation of gluconeogenesis-related enzymes and inhibiting gluconeogenesis via acetylation of PGC-1α and activation of PPARγ. It inhibits glycolysis by inhibiting glycolytic genes (PFK-1, GLUT-1, PGK-1) via inhibition of HIF-1α.

Congestive heart failure and coronary artery diseases are the two most common types of cardiac complications associated with Sirt6 activity (Bindu et al., 2016; D’Onofrio et al., 2017; Li et al., 2017; Guo et al., 2019; Yepuri and Ramasamy, 2019). Deficiency of Sirt6 in cardiomyocytes results in the accumulation of lactate due to impaired glucose oxidation, leading to various comorbidities relating to the heart, including heart failure (Khan D. et al., 2018). Sirt6 heterozygous mice were used to show lactic acid accumulation in mice hearts. Sirt6 deficiency increased FOXO1 localization in heart, upregulating PDK4, reducing oxygen consumption and ATP production, thereby demonstrating the protective role of Sirt6 in maintaining cardiac homeostasis (Khan D. et al., 2018). Another factor involved in the development of cardiac disorders is the formation of atherosclerotic plaque. No evidence suggests a direct link between Sirt6 and atherosclerotic plaque formation (Zi et al., 2019). However, Sirt6 deficiency enhances the expression of pro-inflammatory cytokines like Interleukin-1 and transcriptional signaling of NF-KB (Lappas, 2012; Yu et al., 2013), which are indirectly linked to the pathogenesis of atherosclerosis (Xu et al., 2016). In addition, Sirt6 depletion increased expression of ICAM-1 and PAI-1 and upregulation of p21 gene and reduction of eNOS (Figure 3, Schematic representation of molecular mechanism of Sirt6 in various diseases) further leading to atherosclerotic vascular disease (Yepuri and Ramasamy, 2019; Zi et al., 2019). Sirt6 is also involved in the pathogenesis of cardiomyocyte hypertrophy (Yu et al., 2013; Lu et al., 2016; Ravi et al., 2019; Kanwal et al., 2019b), which includes angiotensin-II induced and IGF-Akt signaling induced cardiac hypertrophy (Li et al., 2017). Increased expression of Sirt6 in cardiomyocytes decreased angiotensin-II action on cardiomyocytes (Yu et al., 2013; Zhang et al., 2017; Eguchi et al., 2018; Ianni et al., 2018). Angiotensin-II induced cardiac hypertrophy is blocked by over-expression of nicotinamide mononucleotide adenylyltransferase-II (Nmnat-II) that is responsible for activation of Sirt6 (Vitiello et al., 2017). Another pathway involved in cardiac hypertrophy is mediated through increased activation of IGF-Akt pathway (Yan et al., 2019) (Figure 3: Schematic representation of molecular mechanism of Sirt6 in various diseases), which was shown in Sirt6 deficient mice (Sundaresan et al., 2012; Vitiello et al., 2017).

## Sirt6 in Neurodegenerative Diseases and Brain Aging

Alzheimer’s disease (AD) is involved in neurodegeneration, and characterized by dementia. AD is pathologically characterized by formation of beta amyloid plaques and neurofibrillary tangles known as tau proteins (Kocahan and Doğan, 2017; Takahashi et al., 2017). Oxidative stress, cell senescence, and aging are the major risk factors for the progression of this disease (Markesbery, 1999; Huang et al., 2016; Kerchner and Wyss-Coray, 2016; Xia X. et al., 2018; Butterfield and Boyd-Kimball, 2018; Trevisan et al., 2019). During the pathogenesis of AD, telomere length has a causal role. As cells divide, telomere length shortens, and this shortening is associated with cognitive impairment, amyloid plaque deposition and hyper-phosphorylation of tau protein that are characteristics of AD (Cai et al., 2013; Forero et al., 2016; Liu et al., 2016). Oxidative stress affects telomeres as they contain guanine, which undergoes oxidation to produce 8-oxo-7,8-dihydro-2-deoxyguanosine (8-oxodG) (Kawanishi and Oikawa, 2004; Cai et al., 2013). Sirt6 maintains telomere function as it prevents telomere dysfunction through WRN protein stabilization at telomeric chromatin (Mohamad Nasir et al., 2018). In addition to telomere maintenance, Sirt6 regulates tau protein stabilization during AD oxidative stress. Activation of Sirt6 maintains both genomic stability in the brain and leads to loss of tau protein stability via inhibition of GSK3α/β (Kaluski et al., 2017; Stein and Toiber, 2017; Kim H. et al., 2018). Sirt6 reduction alters DNA repair in the brain of an AD mouse model, and Sirt6 overexpression prevents Amyloid beta protein (Aβ42) induced DNA damage, thereby proving the beneficial effects of Sirt6 in AD (Jung et al., 2016; Tang, 2017). This is confirmed by the fact that the levels of Sirt6 are reduced in the brains of both AD containing mice as well as AD patients. In addition, Aβ42 decreased Sirt6 levels (Kaluski et al., 2017; Cacabelos et al., 2019). Sirt6 is associated with age related disorders. Activation of Sirt6 is protective in AD and other neurodegenerative disorders involving brain aging, thus proving to be an essential therapeutic target in the treatment of neurodegenerative disorders (Naiman and Cohen, 2018).

## Sirt6 in Lipid Metabolism

Lipid metabolism disorders are pervasive and play a vital role in the pathogenesis of atherosclerosis, leading to cardiovascular diseases (Parhofer, 2016; Schofield et al., 2016). Recently Sirt6 has been shown to have a critical role in lipid metabolism (Ye et al., 2017; Assadi-Porter et al., 2018; Kuang et al., 2018) as it is involved in the regulation of fatty acid synthesis, triglyceride synthesis, cholesterol synthesis, fatty acid beta oxidation, lipolysis, adipogenesis and thermogenesis (Liu et al., 2012; Elhanati et al., 2013; Tao et al., 2013a; Tao et al., 2013b; Chen et al., 2017; Yao et al., 2017; Kuang et al., 2018; Gao et al., 2019). Lipid homeostasis and cholesterol biosynthesis are regulated by a transcription factor called Sterol regulatory binding proteins (SREBP), and the expression of SREBP is controlled by Sirt6 (Eberle et al., 2004). SREBP activation is also implicated in inflammation, autophagy, endoplasmic reticulum stress (Shimano and Sato, 2017). Sirt6 inhibits the expression of SREBP, which is indirectly linked with cholesterol biosynthesis, hence regulating Cholesterol homeostasis (Tao et al., 2013b; Ye et al., 2017). Sirt6 negatively regulates cholesterol biosynthesis by various pathways involving SREBP. It causes downregulation of SREBP by reducing mRNA expression (Kuang et al., 2018). Sirt6 is also recruited to the *Srebp* gene promoter (Figure 3: Schematic representation of molecular mechanism of Sirt6 in various diseases) by FOXO3, where it deacetylates H3K9 and H3K56 in the promoter regions of *Srebp* and suppresses the transcription levels of *Srebp* and its target genes (Tao et al., 2013b; Elhanati et al., 2013; Kugel and Mostoslavsky, 2014). It also inhibits SREBP’s subsequent conversion into active forms (Elhanati et al., 2013; Kugel and Mostoslavsky, 2014) and activates AMPK, leading to the phosphorylation and inactivation of SREBP (Kugel and Mostoslavsky, 2014; Kuang et al., 2018). The absence of Sirt6 leads to increased production of Triglycerides due to increased expression of genes involved in Triglyceride synthesis (Ye et al., 2017). The genes responsible for Fatty acid metabolism by beta oxidation is downregulated (Ye et al., 2017; Kuang et al., 2018). Sirt6 also regulates adipogenesis and thermogenesis, it is an essential factor in adipogenesis, by enhancing casein kinase 2 (CK2) activity (Chen et al., 2017; Kuang et al., 2017; Kuang et al., 2018). Similarly, expression of PGC-1α is a central regulator in thermogenesis. Overexpression of PGC-1α causes mitochondrial oxidative phosphorylation and expression of thermogenic genes. Sirt6 controls expression of PGC-1α, as depletion of Sirt6 decreases the appearance of PGC-1α, thus resulting in decreased thermogenesis (Yao et al., 2017; Kuang et al., 2018; Singh et al., 2020).

## Sirt6 and Diabetes

The prevalence of Type 2 Diabetes Mellitus (T2DM) is increasing at an alarming rate worldwide. With an increased understanding of the pathogenesis of T2DM various new therapeutic approaches are being developed to target the fundamental cause of T2DM, Sirt6 being one among them (Kitada et al., 2013; Bae, 2017; Chellappan et al., 2018). The pathophysiology of T2DM is characterized by many causes (Mahler and Adler, 1999). The primary cause of T2DM is decreased sensitivity of beta cell functioning to the levels of glucose, but recent studies have proven the crucial role Sirt6 in glucose stimulated insulin secretion (GSIS), enhancing the release of insulin (Mahler and Adler, 1999; Song M. Y. et al., 2016; Xiwen et al., 2016) (Figure 3: Schematic representation of molecular mechanism of Sirt6 in various diseases). Various studies show different mechanisms of how Sirt6 is involved in increasing beta cell function. One such pathway is Sirt6 suppresses expression of the thioredoxin-interacting protein (TXNIP), which is engaged in β-cell apoptosis (Shalev, 2014; Qin et al., 2018). Thus, Sirt6 maintains the functioning of beta cells. Another pathway where Sirt6 supports GSIS functioning of beta cells is via regulation of FOXO1 expression. Sirt6 inhibits FOXO1, maintaining the glucose-sensing ability of pancreatic β-cell and systemic glucose tolerance (Song M. Y. et al., 2016). Hence Sirt6 has proven to be a chief regulator in glucose homeostasis (Zhong et al., 2010; Gertman et al., 2018). Studies done on mice have been conclusive in showing that knockdown of Sirt6 can lead to complications, namely severe hypoglycemia leading to death (Lee et al., 2017; Kuang et al., 2018). The primary reason for this were increased uptake from the muscle and adipose tissue rather than intestinal uptake of glucose or increased secretion from the kidneys (Kuang et al., 2018). There is both *in vitro* and *in vivo* evidence, showing that increased glucose uptake may be due to deficiency of Sirt6 (Zhong et al., 2010; Zhong and Mostoslavsky, 2010). Sirt6 suppresses HIF1α (hypoxia inducible factor-1α), which is responsible for suppressing several genes like GLUT-1, LDH, and PDK-1, which coordinate various processes involving glucose metabolism such as glycolysis (Kim et al., 2006; Zhong et al., 2010; Laemmle et al., 2012; Khan D. et al., 2018; Kuang et al., 2018). Growth hormone and IGF-1 signaling alter the metabolism of glucose (Sundaresan et al., 2012; Takasaka et al., 2014). Sirt6 controls gluconeogenesis by the receptor PGC-1α and p53/FOXO1 signaling. Inhibition of PGC-1α activity by Sirt6 occurs via deacetylation of GCN5, increasing its acetyltransferase activity, which is a form of histone acetylation and thereby increases the acetylation of PGC-1α which leads to inhibition of hepatic gluconeogenesis and thereby hyperglycemia (Figure 3: Schematic representation of molecular mechanism of Sirt6 in various diseases) (Jeninga et al., 2010; Satoh and Imai, 2014; Sharabi et al., 2017; Kanwal and Dsouza, 2019; Singh et al., 2019).

## Sirt6 Modulators

**FIGURE 4:**
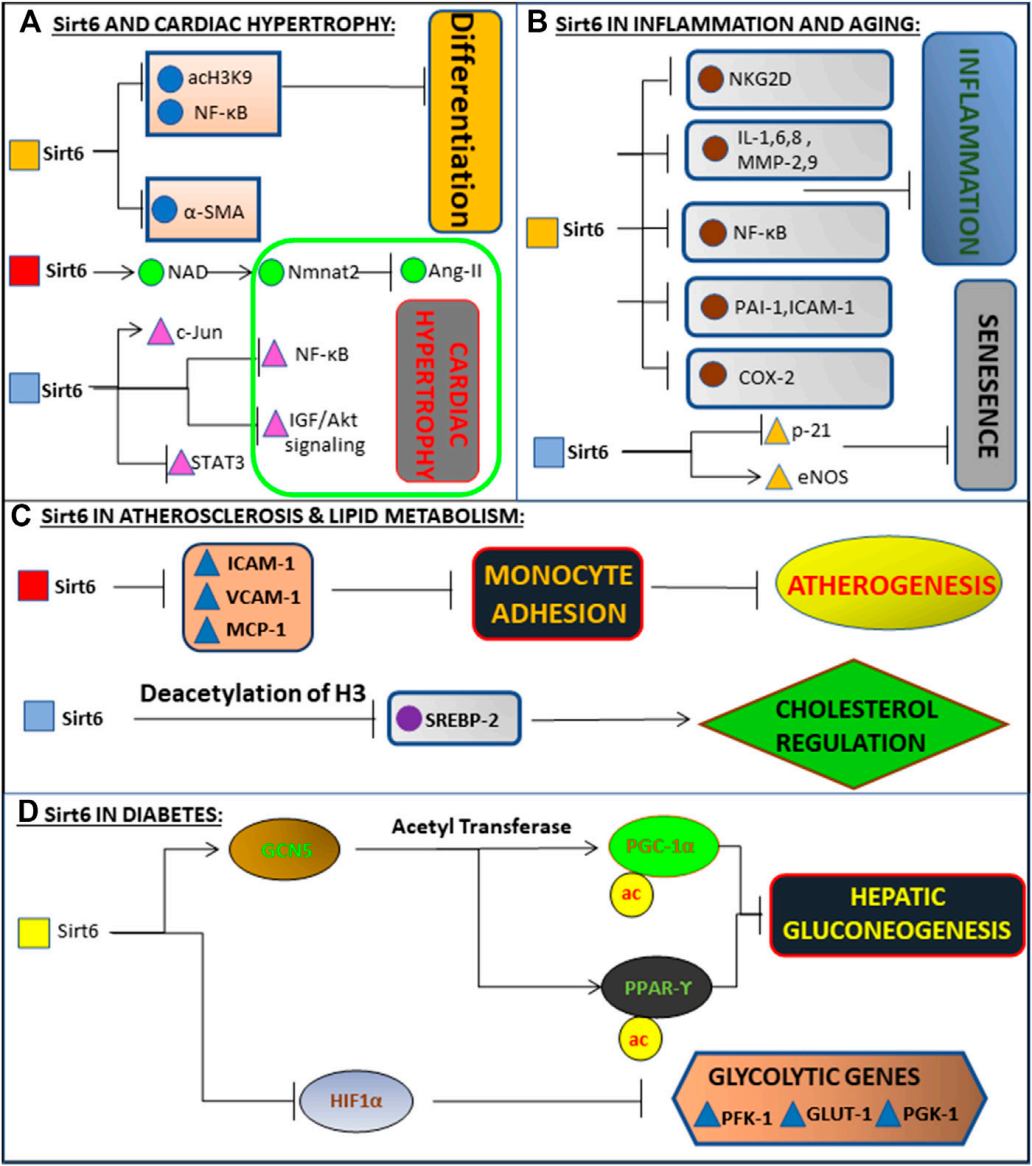
Modulators of Sirt6.

Sirt6 has a plethora of biological activities, making it a vital molecule, allowing researchers to identify Sirt6 modulators (Figure 4, Modulators of Sirt6) in developing effective therapeutic approaches to a broad spectrum of diseases. Some endogenous activators of Sirt6 include Lamin A and long chain free fatty acids (Feldman et al., 2013; Rahnasto-Rilla et al., 2016; Ghosh, 2019). Lamin A increases the deacetylation activity of Sirt6 and Sirt1 by directly interacting with the deacetylating proteins (Ghosh et al., 2013; Ghosh et al., 2015). Free fatty acids were also seen to increase the deacetylate ing activity of Sirt6. Free fatty acids stimulated Sirt6 deacetylase activity, where acyl group binding pocket binds to free fatty acids, splaying Sirt6 subdomains, thus stimulating deacetylase activity (Feldman et al., 2013). In addition to the above endogenous activators, Sirt6 is activated by CR (Caloric restriction), c-Fos protein, p53 and increased intracellular levels of NAD^+^ (Kugel and Mostoslavsky, 2014; Zhang et al., 2014; Zhang et al., 2016b; Li M. et al., 2018; Khan R. I. et al., 2018; Kuang et al., 2018; De Céu Teixeira et al., 2019). Although CR and NAD + activates all isoforms of sirtuins, free-fatty acids and c-Fos protein selectively activates Sirt6. Furthermore, other endogenous activators that indirectly activate Sirt6 include increased Sirt1, FoxO3a, and Nrf-1 (Tosato et al., 2007; D’Onofrio et al., 2017). Pyrrolo (1,2-α) quinoxaline derivatives are among the first discovered Sirt6 activators (Hassanieh and Mostoslavsky, 2018). UBCS039 is another activator of Sirt6 which is one of the first synthetic and specific activators of the same. It has been known to cause Sirt6 specific Histone H3 deacetylation and accentuate autophagy in various types of cancer cells thereby showing its tumor suppressor effects (Iachettini et al., 2018). Another novel Sirt6 activator, CL5D also regulates the process of Histone deacetylation but its exact mechanism and clinical relevance has not yet been clearly elucidated (Klein et al., 2020).

Polyphenols, such as quercetin and luteolin at higher concentrations activate Sirt6, whereas inhibit at lower levels. Thus polyphenols modulate Sirt6 activity in a concentration dependent manner (Rahnasto-Rilla et al., 2016; Rahnasto-Rilla et al., 2018; Heger et al., 2019). Cyanidin, another polyphenol, is a potent activator of Sirt6 producing 5–15 folds increase in Sirt6 activity compared to other polyphenols (Rahnasto-Rilla et al., 2018). These compounds are non-specific modulators of Sirt6, and are able to modulate the activities of other sirtuin isoforms. Selective small molecule activators of Sirt6 like MDL-800 bind to the allosteric site, increasing Sirt6’s deacetylase activity (Klein et al., 2017; You et al., 2017; Huang et al., 2018). This binding led to a significant and overall increase in deacetylation of H3K9ac and H3K56ac in HCC (human-hepatocellular-carcinoma cells) which prevented the proliferation and differentiation of HCC cells through cell cycle arrest thus proving that the activation of Sirt6 is crucial in the treatment of Hepatocellular Carcinoma (Huang et al., 2018). Fluvastatin, which competitively inhibits HMG CoA reductase, reduces cholesterol synthesis and is yet another activator of Sirt6 (Kim J. H. et al., 2018). Exposure of fluvastatin to HepG2 cells increased Sirt6 expression (Kim J. H. et al., 2018; Zhang C. et al., 2019). The mechanism underlying cholesterol regulation when fluvastatin increased Sirt6 expression was via phosphorylation of AMPKα and SREBP-1 pathway (Kim J. H. et al., 2018). Apart from activators, many small molecule Sirt6 inhibitors have been developed over time that directly acts on Sirt6 (Liu and Zheng, 2016). 2,4-dioxo-*N*-(4-(pyridin-3-yloxy) phenyl)-1,2,3,4-tetrahydroquinazoline-6-sulfonamide, a Sirt6 inhibitor, improved glucose tolerance in mice, and reduced insulin, triglycerides, and cholesterol levels indicating that a Sirt6 inhibitor could improve glycemic control in T2DM (Sociali et al., 2017; Khan R. I. et al., 2018). Trichostatin A (TSA) is an inhibitor of Sirt6 that selectively inhibits Sirt6 and no other mammalian sirtuins (Parenti et al., 2014; Wood et al., 2018; You and Steegborn, 2018). A study showed that TSA inhibited Sirt6 thus inhibiting deacetylation of p53 at lysine 382 (Wood et al., 2018), thus providing a lead compound in development of Sirt6 specific inhibitors in regulating apoptosis and stress resistance (Zhao et al., 2019). In addition to the above inhibitors, specific peptides and pseudo peptides including SDK (thioAc)TM^21^, HKK(thioAc)LM^21^, AKK(thioAc)LM^21^ were also studied for their ability to inhibit Sirt6 activity (Kokkonen et al., 2012; Rahnasto-Rilla et al., 2018). Derivatives of Quinazolinedione were recently discovered to inhibit Sirt6 activity, and these compounds were seen to sensitise the tumour cells to chemotherapeutic agents (Sociali et al., 2015; Rahnasto-Rilla et al., 2018). Their activity of inhibition of Sirt6 would have represented a potential therapeutic approach in the treatment of cancer, but this is not entirely true because Sirt6 acts as a double-edged sword in cancer, as activation of Sirt6 has shown to act as a tumor suppressor in many forms of cancer such as Colon, Ovarian, Prostate, Breast Cancer etc. (Lerrer et al., 2015; Lee et al., 2016; Xia Y. Q. et al., 2018) Although various Sirt6 modulators show a promising therapeutic intervention, Sirt6 under certain circumstances seems to play contradictory roles, the reason for this discrepancy is unknown, hence making it difficult to develop Sirt6 modulators (Gomes, Leal et al. 2019).

## Conclusion

In an era where the sheer number of cases of metabolic and cardiovascular diseases is progressively escalating, resulting in a significant health challenge. Scientists are on a continuous search to discover novel molecular targets. One such target is Sirt6, an NAD + dependent histone deacetylase that regulates the expression of several essential genes. As a regulator of gene expression, Sirt6 has been implicated in cancer, neurodegenerative diseases, heart diseases, diabetes, and aging-related processes. Popularly known as “longevity protein,” Sirt6 plays a critical role in aging by controlling cellular processes including genomic stability, DNA-repair, maintenance of telomere length, thereby increasing lifespan. Sirt6 involvement in increasing lifespan requires a better understanding of the molecular mechanisms of Sirt6 in humans relating to aging. In cardiovascular diseases, Sirt6 is involved in regulating the heart's multiple pathophysiological conditions, including hypertrophy, atherosclerotic vascular disease, coronary artery disease, and heart failure. Abnormal lipid metabolism also plays a crucial role in the pathogenesis of heart disease. Sirt6 activation is beneficial in alleviating various factors involved in heart disease. The ubiquity of neurodegenerative diseases is increasing, and decreased levels of Sirt6 in degenerative disease animal models have given a clear understanding of its role, especially in AD. The purpose of Sirt6 in T2DM has been extensively studied, as it improves major pathophysiological defects in pancreatic β-cells, skeletal muscle, and tissues impaired during T2DM. Therefore, Sirt6 plays a crucial role in metabolic diseases and neurodegenerative diseases; future studies should be directed in developing genetic and pharmacologic activation of Sirt6. Various activators and inhibitors that directly and indirectly modulate the activity of Sirt6 have been discovered. Further studies on these Sirt6 modulators may generate potential therapeutic targets that may enhance therapy. Sirt6 has the potential to be a critical therapeutic target in clinical approaches to treating a broad spectrum of disease states. Due to the diversified effects of Sirt6, it poses both a challenge as well as a ray of hope for extensive studies to understand its precise mechanism and functioning, further as a potential therapeutic target, it can enhance or even substitute existing lines of therapy. Apart from the development of new modulators, extensive research must be done in concluding the roles of Sirt6 in various other diseases.

## Author Contributions

AK and SPS has designed, edited, and revised the manuscript. SR and LAD have written the manuscript. Final proof reading is done by by SPS and AK. All the authors have agreed the ultimate version.

## Conflict of Interest

The authors declare that the research was conducted in the absence of any commercial or financial relationships that could be construed as a potential conflict of interest.
